# Authors’ response: Absence of cross-reactivity of the cobas WNV assay with human pegiviruses

**DOI:** 10.2807/1560-7917.ES.2025.30.21.2500326

**Published:** 2025-05-29

**Authors:** Dániel Cadar, Jonas Schmidt-Chanasit, Annette Reissinger, Stefano Orru', Sarah Anna Fiedler, Markus Benedikt Funk

**Affiliations:** 1 Department of Arbovirology and Entomology, National Reference Center for Tropical Infectious Pathogens, Bernhard Nocht Institute for Tropical Medicine, Hamburg, Germany; 2 Faculty of Mathematics, Informatics and Natural Sciences, University of Hamburg, Hamburg, Germany; 3 Section of Molecular Virology, Paul-Ehrlich-Institut, Langen, Germany; 4 Division of Safety of Biomedicines and Diagnostics, Paul-Ehrlich-Institut, Langen, Germany

**Keywords:** Cross-reactivity, Pegiviruses, cobas WNV Assay, Blood donor screening

**To the editor: **We thank Saa et al. for the thoughtful review of our publication ‘Assessment of the effectiveness of West Nile virus screening by analysing suspected positive donations among blood donors, Germany, 2020 to 2023’ [1] and the insightful comments regarding the sensitivity of the cobas West Nile virus (WNV) assay and the interpretation of our metagenomic next-generation sequencing (mNGS) findings.

We would like to address and clarify the main points raised:

We fully acknowledge, as discussed in our article, that the cobas WNV assay has a superior analytical sensitivity expressed as limit of detection (LoD; ca 6–13 copies/mL) compared with mNGS (ca 10^3^ copies/mL). In our study, mNGS was employed to support the identification and lineage determination of WNV in suspected positive blood donations, as well as to detect alternative viral pathogens. However, because of its lower sensitivity compared with nucleic acid amplification technique (NAT)-based assays and potential RNA degradation during storage of blood samples, mNGS findings were interpreted cautiously: a positive mNGS result confirmed WNV infection whereas a negative mNGS result did not exclude low-level WNV viraemia. Therefore, mNGS was used as an exploratory and supportive tool, but NAT remained the primary method for sensitive detection of low-level WNV RNA.

We appreciate the additional in silico analysis provided regarding the lack of sequence homology between the cobas WNV assay oligonucleotides and human pegivirus (HPgV) genomes. This analysis suggests that direct assay cross-reactivity with HPgV-2 is unlikely. We would like to emphasise that, in our article, we did not conclude that cross-reactivity with HPgV was definitively demonstrated. We suggested that in some suspected cases where WNV was not confirmed, mNGS identified other viruses, including HPgV-2, raising the hypothesis that alternative explanations such as coincidental viraemia could exist. Given the known prevalence of HPgV-2 in blood donors (2–3% in Europe, higher in other regions), and its detection in unrelated blood donor studies, it is indeed plausible that the two HPgV-2 findings in our study reflect coincidental viraemia rather than assay cross-reactivity. 

Our findings reinforce the robustness of NAT-based donor screening as an effective risk minimisation measure for WNV transfusion-transmitted infections. At the same time, Saa et al. highlight the importance of pathogen surveillance efforts in remaining vigilant of emerging or co-circulating viruses in donor populations.

In conclusion, we agree that direct cross-reactivity between the cobas WNV assay and HPgV-2 is highly unlikely based on available data. The mNGS findings of HPgV-2 RNA are best interpreted as coincidental infections in blood donors, independent of the initial WNV NAT-based screening results. We sincerely thank Saa et al. for the constructive input, which allows for a more precise interpretation of our data and strengthens the ongoing scientific dialogue on blood safety and diagnostic assay performance.
